# Lytic Bacteriophage EFA1 Modulates HCT116 Colon Cancer Cell Growth and Upregulates ROS Production in an *Enterococcus faecalis* Co-culture System

**DOI:** 10.3389/fmicb.2021.650849

**Published:** 2021-03-31

**Authors:** Mwila Kabwe, Terri Meehan-Andrews, Heng Ku, Steve Petrovski, Steven Batinovic, Hiu Tat Chan, Joseph Tucci

**Affiliations:** ^1^Department of Pharmacy and Biomedical Sciences, La Trobe Institute for Molecular Science, La Trobe University, Bendigo, VIC, Australia; ^2^Department of Physiology, Anatomy and Microbiology, La Trobe University, Melbourne, VIC, Australia; ^3^Department of Microbiology, Royal Melbourne Hospital, Melbourne, VIC, Australia

**Keywords:** *Enterococcus faecalis*, bacteriophage, genomics, biofilm, colon cancer proliferation, reactive oxygen species

## Abstract

*Enterococcus faecalis* is an opportunistic pathogen in the gut microbiota that’s associated with a range of difficult to treat nosocomial infections. It is also known to be associated with some colorectal cancers. Its resistance to a range of antibiotics and capacity to form biofilms increase its virulence. Unlike antibiotics, bacteriophages are capable of disrupting biofilms which are key in the pathogenesis of diseases such as UTIs and some cancers. In this study, bacteriophage EFA1, lytic against *E. faecalis*, was isolated and its genome fully sequenced and analyzed *in silico*. Electron microscopy images revealed EFA1 to be a *Siphovirus*. The bacteriophage was functionally assessed and shown to disrupt *E. faecalis* biofilms as well as modulate the growth stimulatory effects of *E. faecalis* in a HCT116 colon cancer cell co-culture system, possibly via the effects of ROS. The potential exists for further testing of bacteriophage EFA1 in these systems as well as *in vivo* models.

## Introduction

Understanding the interactions between host and microbe is cardinal to the development of treatment modalities to curb pathogen associated disease. In the human body, the number of bacteria cells is relatively similar to that of human cells (Sender et al., 2016), with the gut being the largest reservoir (Kho and Lal, 2018). *Enterococcus* species which are Gram-positive facultative anaerobes form approximately 1% of all microbiota in the gut and are readily isolated from human and animal feces as well as from the environment (Dubin and Pamer, 2014). Enterococci, including *E. faecium* and *E. faecalis* are the most predominant species and have traditionally been considered normal flora and sometimes used as probiotics to treat gut infections (Franz et al., 2003). Previous recommendations for use as probiotics stemmed from studies that explored the capacity for *Enterococcus* spp. to attach to epithelial cells thereby preventing colonization of more pathogenic bacteria (Kropec et al., 2005; Nueno-Palop and Narbad, 2011; Tinrat et al., 2018; Wang J. et al., 2020). However, emerging trends show that *Enterococcus* spp. are a major cause of a range of difficult to treat nosocomial infections, with implicated isolates displaying both acquired and intrinsic multi-antibiotic resistance (Franz et al., 2003; Hollenbeck and Rice, 2012). *Enterococcus* spp. survive on inanimate objects (e.g., intravenous catheters, handrails and bed frames) as fomites (Zervos et al., 1987; Hota, 2004) creating an opportunity for the transmission of antibiotic resistant Enterococci among patients in hospitals via their attending health workers (Dubin and Pamer, 2014; Drees et al., 2008). Although both *E. faecium* and *E. faecalis* are predominant Enterococci in the normal gut microbiota (De Lastours et al., 2017), the latter has been implicated in a higher frequency of Hospital Acquired Infections (HAI) (Weiner-Lastinger et al., 2020). In the American national surveillance program report from 2015 to 2017 involving more than 2,400 hospitals, *E. faecalis* was the fifth most common cause of HAI after *Escherichia coli*, *Staphylococcus aureus*, *Klebsiella* spp. and *Pseudomonas aeruginosa* while it was the most frequently reported pathogen in oncology units (Weiner-Lastinger et al., 2020).

Although *E. faecalis* has traditionally been considered a normal constituent of the gut microbiome, recent data suggests that it is intrinsically linked to colorectal cancer (CRC) (Lennard et al., 2016). A study quantifying bacterial prevalence in feces of CRC patients found an increased level of *E. faecalis* compared to normal controls (Balamurugan et al., 2008) while transcriptional modeling of host genes expressed in *E. faecalis* colonized CRC showed activation of pathways related to tumor invasion and cancer metastasis (Lennard et al., 2016). Further, *E. faecalis* has been shown to colonize the murine gastrointestinal tract by formation of bacterial biofilms (Barnes et al., 2017), promote aneuploidy and tetraploidy, induce colonic epithelial DNA double-strand breaks and arrest cell cycle (Wang et al., 2008) via increased reactive oxygen species (ROS) production (Huycke et al., 2002; Wang and Huycke, 2007; Wang et al., 2008). The bacterial microbiome is now considered an important aspect of the tumor microenvironment. The recent highlighting of the role of oncobacteria in tumorigenesis has added weight to the argument that tumor microbiota manipulation could be key to unlocking the potential of cancer chemotherapy and immunotherapy (Bhatt et al., 2017; Rajagopala et al., 2017; Helmink et al., 2019; Parhi et al., 2020).

The abundance of bacteria in humans and the environment pales in comparison to their viral predators, the bacteriophages. For instance, bacterial numbers are estimated at 10^11^ cells per gram weight of fecal material compared to approximately 10^12^ bacteriophages in the same amount (Cani, 2018). Bacteriophages have been shown to drive the composition of the gut microbiome (Moreno-Gallego et al., 2019) by specifically targeting gut bacteria and creating an evolutionary arms race between bacterial host and virus (Azam and Tanji, 2019; Wang G. et al., 2020). Further, studies have shown the capacity of bacteriophages to disrupt biofilms (Hansen et al., 2019; Lusiak-Szelachowska et al., 2020), kill intracellular bacteria (Jonczyk-Matysiak et al., 2015), and modulate the immune system (Van Belleghem et al., 2018), all key to counteracting virulence mechanisms that oncobacteria use to promote tumor growth (Holt and Cochrane, 2017). Bacteriophage against *Fusobacterium nucleatum*, a classic oncobacterium implicated in various cancers (Mitsuhashi et al., 2015; Yamamura et al., 2016; Al-Hebshi et al., 2017; Zhao et al., 2017; Yang S. F. et al., 2018; Gaiser et al., 2019; Kharrat et al., 2019; Yachida et al., 2019), have shown the potential to augment chemotherapy in the treatment of *F. nucleatum* associated CRC (Zheng et al., 2019). In this study, we demonstrate the augmentation of CRC cell growth *in vitro* by *E. faecalis*, and how these effects can be modulated by treatment with the *E. faecalis* specific bacteriophage EFA1, possibly via an increase of ROS production. Bacteriophage EFA1 was isolated from wastewater, phenotypically characterized and its whole genome sequenced. The whole genome sequence of EFA1 bacteriophage has been deposited in GenBank ^®^ (Benson et al., 2000) under accession number MT857001.

## Materials and Methods

### Ethics Statement

Study protocols were approved by the La Trobe University Ethics Committee under reference number S17-112 and all methods performed in accordance with the La Trobe University Ethics, Biosafety and Integrity guidelines, and regulations.

### HCT116 Human Colon Cancer Cell Culture and Bacterial Culture Conditions

Human colonic HCT116 cells (ATCC CCL-247) were maintained in RPMI-1640 medium with L-glutamine and sodium bicarbonate (Sigma-Aldrich ^®^, Australia) that was supplemented with 10% fetal bovine serum (FBS; Sigma-Aldrich ^®^, Australia) and passaged by detachment using 0.5% trypsin (Sigma-Aldrich ^®^, Australia). HCT116 cells were maintained in humidified 5% CO_2_ at 37°C.

The *E. faecalis* strains used in these experiments were isolated from wastewater. They were cultured at 37°C in Brain-Heart infusion media (BHI; Oxoid^TM^, Australia) under anaerobic conditions. Anaerobic conditions were generated using the AnaeroGen pack (Oxoid^TM^, Australia). For confirmation of *E. faecalis* strain identity, 16s rRNA PCR gene amplification was performed and the amplicons purified using the QIAquick ^®^ PCR purification kit (Qiagen, Australia) before Sanger sequencing at the Australian Genome Research Facility. The primers used for the 16s rRNA PCR and sequencing were U27F: 5′AGAGTTTGATCMTGGCTCAG3′ and U492R: 5′AAGGAG GTGWTCCARCC 3′ under thermocycling conditions: 95°C for 3 min, 32 cycles of 95°C for 30 s, 60°C for 30 s, and 72°C for 90 s, with a final extension at 72°C for 10 min (Kabwe et al., 2019).

### Bacteriophage Isolation and One-Step Growth Curve Determination

Bacteriophage isolation was carried out as previously described (Kabwe et al., 2020). Wastewater from Victoria, Australia was collected and filtered using 0.2 μm cellulose acetate filters (Advantec, Australia). Filtered wastewater was then added to 10^8^ colony forming units/mL (CFU/mL) of *E. faecalis* in BHI broth at 1% (v/v). The *E. faecalis*-wastewater enrichment culture was then incubated at 37°C anaerobically for 4 d before 0.2 μm cellulose acetate filtration. Serially diluted enrichment filtrates (10 μL) were placed on fresh lawns of *E. faecalis* on BHI containing 1% agar and plates incubated for 24 h. Any potential bacteriophage clearing was excised (along with a portion of agar) and resuspended in 500 μL of media, before centrifugation (12,000**×** *g* for 5 min) and a 10-fold serial dilution was completed. Then 10 μL of each dilution was placed on a bacterial lawn such that plaques could be observed after an overnight anaerobic incubation at 37°C. This serial dilution purification was repeated five times to ensure single virion infection. The concentration of the bacteriophage suspensions was determined and calculated as number of plaque forming units (PFU)/mL.

Host range was assessed by making 10-fold serial dilutions of the bacteriophage stock (1 × 10^8^ PFU/mL) and spotting 10 μL of the serial dilution aliquots onto freshly plated lawns of oral and enteric bacteria including, *F. nucleatum*, *Solobacterium moorei*, *Streptococcus mutans*, *Lactobacillus Casei*, *Aeromonas hydrophila*, and *Escherichia coli.* All plates were incubated at 37°C under anaerobic conditions except for *E. coli* and *A. hydrophila* that were incubated under aerobic conditions. A lack of individual plaques indicated that the bacteriophage EFA1 did not target these strains.

To determine the one-step growth curve (OSGC), 1 mL of *E. faecalis* in exponential growth phase at the concentration of 1 × 10^8^ CFU/mL was centrifuged at 12, 000 × *g* for 5 min. The cell pellet was then resuspended in 900 μL of cold BHI broth before adding 100 μL of EFA1 bacteriophage at multiplicity of infection (MOI) of 0.1 as previously described (Kabwe et al., 2020). The bacteria-bacteriophage mixture was incubated at 4°C for 5 min to allow for bacteriophage adsorption onto bacteria. The mixture was then centrifuged at 12,000 × *g* for 10 min and unadsorbed bacteriophages assayed. Adsorbed bacteriophage were together collected with bacteria in a pellet and resuspended in BHI broth at 50 × dilution, incubated anaerobically at 37°C and bacteriophage concentration determined every 5 min. A graph of bacteriophage concentration (*y*-axis) against time (*x*-axis) was plotted and burst size (PFU/bacterial cell) calculated as a fraction of burst of newly released bacteriophage out of the total number of infecting bacteriophages.

### Transmission Electron Microscopy

Visualization of the bacteriophage phenotype was achieved using a JEOL JEM-2100 transmission electron microscope (TEM) operated at 200 kV as previously described (Kabwe et al., 2020). Using a 400-mesh formvar and carbon copper grids (ProScieTech, Australia), EFA1 bacteriophage particles were adsorbed for 1 min before rinsing the grids with milli-Q water and negatively staining for 20 s with 2% (w/v) uranyl acetate (Sigma-Aldrich^®^, Australia). Excess uranyl acetate was removed, and the copper grids allowed to air dry at room temperature for 30 min. The Gatan Orius SC200D 1 wide-angle camera coupled to the Gatan Microscopy Suite and Digital Micrograph Imaging software (Version 2.3.2.888.0) was used to take the TEM images before being exported to Image J (Version 1.8.0_112) for further analysis.

### Bacteriophage DNA Extraction

Using an established method (Kabwe et al., 2019), 10 mL of 1 × 10^8^ PFU/mL bacteriophage solution in phosphate buffered saline (PBS, pH 7.4) was prepared for genomic DNA extraction. In brief, the bacteriophage solution was treated for 30 min at room temperature with 5 mmol/L of MgCl_2_ (Sigma-Aldrich^®^, Australia), and 10 μg/mL RNAse A and DNAse I (Promega, Australia). Bacteriophage particles were then precipitated at 4°C with 10% (w/v) Polyethylene glycol (PEG-8000) and 1 g/L sodium chloride, and resuspended in 50 μL nuclease-free water (Promega, Australia) before viral proteins were digested with 50 μg/mL of proteinase K, 20 mmol/L EDTA (Sigma-Aldrich^®^, Australia) and 0.5% (v/v) of sodium dodecyl sulfate (Sigma-Aldrich^®^, Australia) for 1 h at 55°C. An equal volume of phenol-chloroform-isoamyl alcohol (29:28:1) (Sigma-Aldrich^®^, Australia) was added to the DNA/bacteriophage protein mixture and their columns separated by centrifugation at 12,000 × *g* for 10 min to collect an aqueous phase with bacteriophage DNA. The bacteriophage DNA was then precipitated out using 70% ethanol and collected by 12,000 × *g* centrifugation before resuspension in 30 μL nuclease-free water (Promega, Australia).

### Bacteriophage Whole Genome Sequencing and *in silico* Analysis

Bacteriophage whole genome sequencing was performed on the Illumina MiSeq^®^ technology using the NEBNext^®^ Ultra^TM^ II DNA Library Prep Kit (NEB) and a MiSeq^®^ V3 600 cycle reagent kit (Illumina, Australia) according to manufacturer’s instructions. Generated reads were trimmed using Trim Galore v0.6.4 with the default settings (Q scores of ≥ 20, with automatic adapter detection) and assembled using the Unicycler *de novo* assembly pipeline (Wick et al., 2017). PhageTerm was used to reorient the phage genome to begin at its pac site (Garneau et al., 2017) before exporting to Geneious (Version 11.0.5)^1^. Translated open reading frames (ORFs) were mapped onto the National Centre for Biotechnology Institute (NCBI) database using BLASTP (Mount, 2007) and annotated sequence submitted to GenBank^®^. To allocate taxa for bacteriophage EFA1, Viral Proteomic Tree (ViPTree) webserver was used to construct a viral proteomic tree with other related bacterial viral genomes in the reference database (Nishimura et al., 2017). The generated proteomic tree was annotated using the Interactive Tree of Life (iTOL) (Letunic and Bork, 2019). Amino acids of putative beta-lactamase protein in bacteriophage EFA1 and bacteria (*E. faecalis*) were aligned by a pair-wise progressive alignment in CLC genomics workbench version 9.5.4.

### Biofilm Growth Analysis

The *E. faecalis* mono-biofilms were grown in 96 well polystyrene plates as described previously (Kabwe et al., 2020). Briefly, 100 μL of 10^8^ CFU/mL *E. faecalis* in BHI broth supplemented with 5% glucose (Sigma-Aldrich^®^, Australia) was added to sterile BHI (100 μL) and incubated anaerobically for 4 d at 37°C and 120 rpm shaking (Ratek Medium Orbital shaking incubator). Bacteriophage was added at concentration 10^8^ PFU/mL and biofilm bacteriophage mixture allowed to incubate anaerobically at 37°C. The biofilm mass was determined at 2, 4, 6, 8, and 24 h post bacteriophage treatment (Merritt et al., 2005). Biofilms were rinsed in milli-Q water for 5 min and stained with 200 μL of 1% aqueous crystal violet for 10 min. Excess crystal violet was rinsed off in milli-Q water. Adherent crystal violet was solubilized in 70% ethanol and absorbance read on the FlexStation 3 plate reader (Molecular Devices, United States) at 600 nm wavelength.

In order to visualize the *E. faecalis* biofilm and the disruptive effects of bacteriophage EFA1, the biofilms were cultured on glass slides and stained with SYBR^®^ gold (Eugene, United States) and Propidium Iodide (PI), each at final concentration of 2 μg/mL. The biofilms were visualized on the Olympus Fluoview Fv10i-confocal laser-scanning microscope (Olympus Life Science, Australia). The excitation wavelength was 485 nm, and emission wavelengths of 535 nm (green) and 635 nm (red) to indicate membrane intact and compromised cells, respectively.

### HCT116 Colon Cancer Cell, Bacteria, and Bacteriophage Co-culture System

For the HCT116 and *E. faecalis* co-culture experiments, *E. faecalis* in exponential growth phase was resuspended in RPMI 1640 media with 10% FBS (Sigma-Aldrich, Australia) before adding to a suspension of HCT116 cells at MOI of 10. For treatment with bacteriophages, a single plaque was purified and concentrated to make a working stock that was further purified by removing lipoteichoic acid and other cell debris as performed by Branston and colleagues (Branston et al., 2015) with minor modification. Briefly, after treating with 10 μg/mL DNase I and RNase A to digest any naked nucleic acids, bacteriophage was precipitated in PEG and NaCl at 4°C for 10 min. Precipitated bacteriophage particles were treated with 2% v/v Triton^®^, Australia) and washed 3 times in PBS (pH 7.4) by centrifugation at 12,000 × *g* for 15 min. The precipitation, Triton^®^ X-100 treatment and PBS (pH 7.4) wash steps were repeated 3 times before precipitated bacteriophage was resuspended in RPMI 1640. *E. faecalis* present in HCT116 co-cultures were treated with the bacteriophage at an MOI of 0.1 and incubated for 48 h in humidified 5% CO_2_ at 37°C.

### HCT116 Cancer Cell Proliferation Assay

Colon cancer cells and co-cultures were incubated at 37°C for 48 h in humidified 5% CO_2_ and proliferation determined by Sulforhodamine B (SRB) assay (Skehan et al., 1990). Briefly, cells were fixed in 10% (w/v) trichloroacetic acid (Thermo Fisher Scientific, Australia) for 30 min at 4°C. Fixed cells were then washed in milli-Q water 5 times before staining with 1% (w/v) SRB (Sigma-Aldrich^®^) in glacial acetic acid (Thermo Fisher Scientific, Australia). Excess SRB was washed off in 1% (v/v) glacial acetic acid before using 10 mM of unbuffered TRIS base to bring the cell associated SRB into solution. Absorbance at 540 nm was measured using the FlexStation 3 plate reader (Molecular Devices, United States).

### Reactive Oxygen Species Determination

Reactive oxygen species activity was evaluated using the ROS-Glo^TM^ Hydrogen Peroxide (H_2_O_2_) assay kit (Promega, Australia), according to the manufacturer’s instructions. Briefly, 48 h co-cultures in 96-well plates were treated with H_2_O_2_ substrate solution at a final concentration of 25 μM. The treated co-cultures were then further incubated at 37°C, 5% CO_2_ for 6 h before adding an equal volume of ROS-Glo^TM^ detection solution. This was then incubated for 20 min before reading relative luminescence units (RLU) on the FlexStation 3 plate reader (Molecular Devices, United States).

### Statistical Analysis

To assess bacteriophage capacity to disrupt biofilms and to modulate *E. faecalis* effects on the colon cancer cell line, Shapiro-Wilk was used to test for the normality of the data. For data that were determined to be normally distributed, the means between two groups were compared using paired *T*-test whilst one-way analysis of variance (ANOVA) was used to compare means of more than two groups. Data on biofilms and cell proliferation were normally distributed while luminescence data were not. In this case, median of two different groups were compared using the Mann-Whitney U test. All data were visualized as box plots illustrating the five-number statistic comprising median, 25th, 75th percentile and upper (Q3 + 1.5 × IQR) and lower limit (Q1-1.5 × IQR). All statistical analysis was performed using the Statistical Package for Social Sciences (SPSS, Inc., United States) with *P*-values less than 0.05 considered statistically significant.

## Results

### Isolation and Phenotypic Characterization of *E. faecalis* Bacteriophage EFA1

**FIGURE 1:**
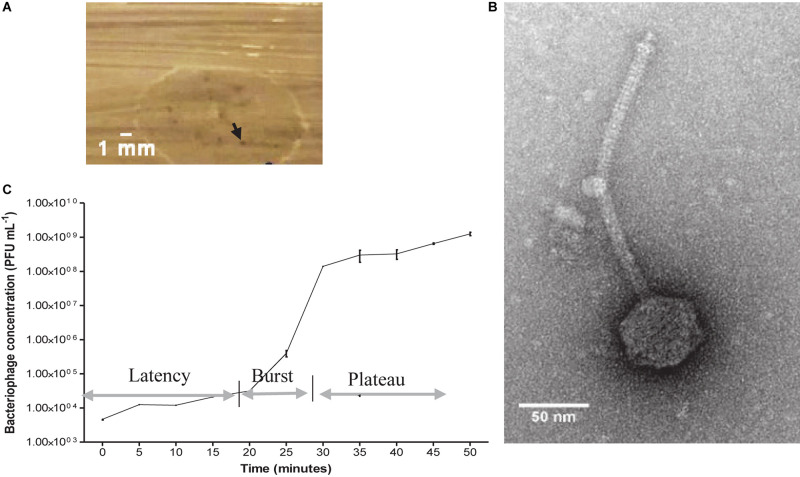
EFA1 plaque morphology, TEM image and growth kinetics. Pin prick plaques with diameter approximately 0.3 mm on *E. faecalis* host **(A)**. TEM revealed *Siphoviridae* morphology with capsid diameter of 58 ± 3 nm and tail length of 223 ± 7 nm **(B)**. EFA1 OSGC: latent phase of 20 min; burst lasting 10 min; plateau phase occurs after approximately 30 min **(C)**.

After combining the filtrate of the wastewater and *E. faecalis* 4 d enrichment, pinprick-sized plaques were visualized on agar plates. Image analysis revealed plaques to be approximately 0.3 mm in diameter (Figure 1A). A concentrated bacteriophage stock assessed by TEM indicated *Siphoviridae* viral particles with a tail length approximately 223 ± 7 nm and capsid diameter approximately 58 ± 3 nm (Figure 1B). The host range of EFA1 extended to both of the *E. faecalis* strains isolated in this study but not other oral/enteric bacteria such as *F. nucleatum*, *Solobacterium moorei*, *Streptococcus mutans*, *Lactobacillus Casei*, *Aeromonas hydrophila*, or *Escherichia coli*. The OSGC analysis revealed EFA1 on the host *E. faecalis* to have a latent period of 20 min. Initial release up to the point where no new bacteriophage were released (burst) took 10 min with each bacterium releasing 120 plaque forming units (PFU)/cell (Figure 1C).

### EFA1 Whole Genome Analysis

**FIGURE 2:**
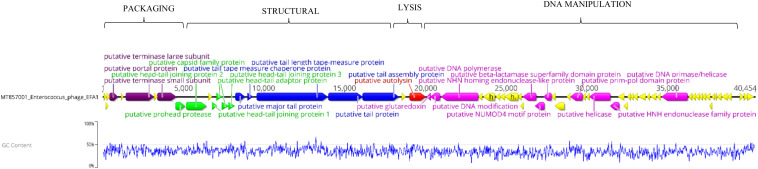
Bacteriophage EFA1 genome orientated according to bacteriophage termini (PAC site). Putative genes coding for DNA packaging (purple), capsid genes (green), tail genes (blue) and putative endolysin (red) are orientated in the left-to-right direction, while putative DNA manipulation genes (pink) are in the opposite direction. Other putative genes whose functionality could not be predicted were considered hypothetical genes (yellow).

**FIGURE 3:**
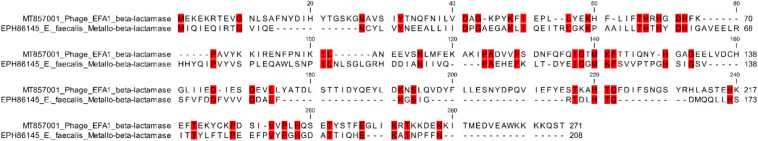
Amino acid alignment of the *E. faecalis* beta-lactamase protein (EPH86145) with EFA1 bacteriophage putative beta-lactamase protein (ORF number 33). Red colored regions highlight areas of identical amino acids [47/272 (17.3%)] between the bacterial and bacteriophage putative beta-lactamase proteins, whereas the non-colored areas define regions where amino acids were non-identical.

Whole genome sequencing revealed a linear dsDNA genome of 40,454 bp with GC content of 34.8% and 68 predicated ORFs. The ORFs were bi-directional with the predicted genes encoding packaging, structural and lysis putative proteins orientated from left to right and putative genes encoding DNA manipulation functions orientated in the opposite direction (Figure 2). After arrangement of the genome so that the putative terminase gene marked the start (Garneau et al., 2017), putative terminase and portal proteins were followed by putative capsid genes then genes predicated to connect the capsid to tail and putative tail genes. The putative autolysin was located after the putative tail genes and was the last gene in the left-to-right orientation, before putative glutaredoxin in the opposite direction (Figure 2). The putative Glutaredoxin was then followed by putative DNA modification, DNA polymerase and interestingly, a putative beta-lactamase superfamily domain in ORF 33 (Figure 2). However, alignment of the putative protein sequence of ORF 33 and the *E. faecalis* metallo-beta-lactamase protein, revealed low homology (Figure 3). Analysis of the EFA1 genome using InterProScan (Mitchell et al., 2019) and Prosite (Sigrist et al., 2013) did not reveal any active sites or beta-lactamase domains required for catalysis (Moali et al., 2003). These findings were confirmed by the Comprehensive Antibiotic Resistance Database (CARD) (Jia et al., 2017) program, which predicted that the EFA1 genome did not carry any functional antibiotic resistance genes. The EFA1 genome also contained genes coding for putative DNA manipulation enzymes including putative endonucleases, NUMOD4 motif protein, helicase and primase. Putative hypothetical genes made up 57.4% (39/68) of EFA1 ORFs. There were no tRNAs, tmRNAs (Laslett and Canback, 2004; Lowe and Chan, 2016), CRISPR sequences (Grissa et al., 2007), or genes predicted to code for integrase found in the bacteriophage EFA1 genome.

### Bacteriophage Phylogeny

**FIGURE 4:**
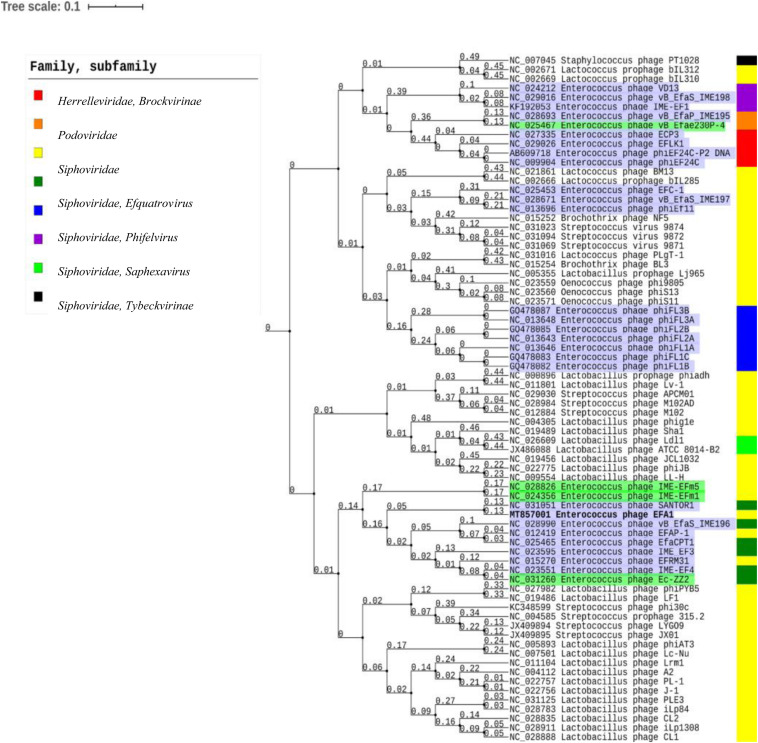
Proteomic tree showing the genome-wide proteomic diversity between bacteriophage EFA1 (highlighted in bold font) and related bacteriophage genomes. Bacteriophages targeting *E. faecalis* are highlighted in purple while those targeting *E*. *faecium* are highlighted in green. Color coded bar represents the family and subfamily of the bacteriophage (key is at top left).

Using the ViPTree server, bacteriophages that clustered with EFA1 were selected to generate a proteomic tree based on genome wide sequence similarities. This was comprised of bacteriophages targeting *Enterococcus, Lactobacillus, Streptococcus, Oenococcus*, and *Staphylococcus* bacteria. Proteomic analysis revealed that EFA1 is part of a monophyletic group with the *E. faecalis* bacteriophage SANTOR1 (Subfamily *Efquatrovirus*), with which it is most closely related (nucleotide similarity of 93.42% over 79% of the whole genome and 87% amino acid similarity). These two are part of a clade of 11 *Enterococcus* bacteriophages which target *E. faecalis* or *E. faecium*, and which also form a larger clade with bacteriophages infecting *Lactobacillus* and *Streptococcus* hosts, but cluster more distantly from other *Siphoviridae*, *Herelleviridae* and *Podoviridae* bacteriophages which target *E. faecalis* hosts (Figure 4).

### Disruption of *E. faecalis* Mono-Biofilm by Bacteriophage EFA1

#### Viability of Biofilm

**FIGURE 5:**
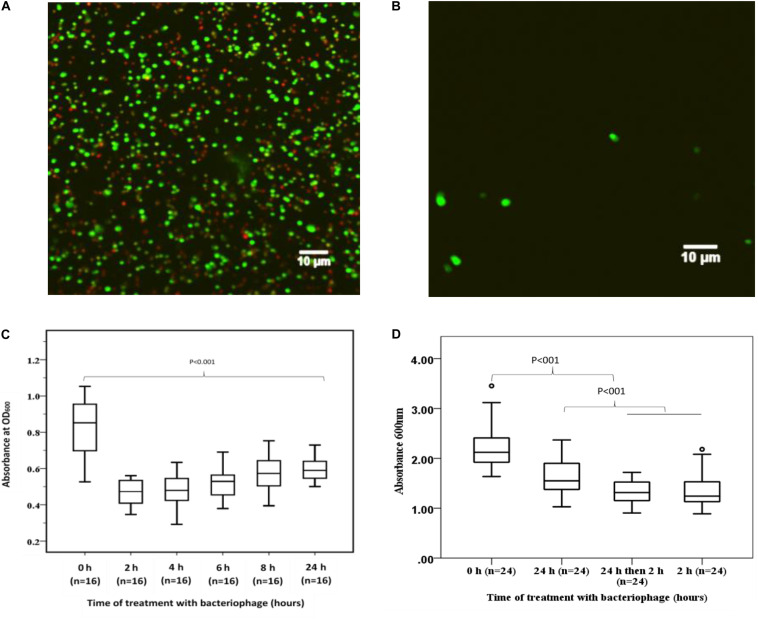
Effects of EFA1 bacteriophage on *E. faecalis* biofilm viability and mass. **(A)** Untreated *E. faecalis* biofilm showing bacteria attached to slide were predominantly membrane intact cells (green), with some bacteria with leaky membranes (red/brown). **(B)** Bacteriophage EFA1 treated biofilm with scant cells remaining attached to slide. **(C)** Quantification of biofilm at time 0 h (without EFA1 treatment) is significanlty greater than any other biofilm treated with bacteriophage EFA1. The lowest amount of biofilm mass was seen after 2 h treatment. Biofilm mass steadly increased with exposure to EFA1. **(D)** 5 days biofilm treated with EFA1 for 24, 2, and 24 h + 2 h, showing similar disruption at 24 + 2 h exposure as that for 2 h.

To demonstrate the capacity of bacteriophage EFA1 to disrupt *E. faecalis* biofilms, confocal imaging was used to visualize biofilm mass on glass slides. Untreated *E. faecalis* biofilm mass (Figure 5A) and that with 2 h bacteriophage EFA1 treatment (Figure 5B) were stained with SYBR Gold^®^ and PI to indicate membrane intact bacterial cells (green color) as proxy for live cells and dead/membrane compromised cells indicated by the red color. Images indicated a densely populated biofilm mass in the untreated control (Figure 5A) with both live and dead cells remaining attached to the glass slide while a sparse population of membrane intact bacteria remained on the EFA1 treated biofilm (Figure 5B).

#### Quantification of Biofilm Mass

In order to quantify the effect of bacteriophage EFA1 on *E. faecalis* mono-biofilms, these were grown in polystyrene 96 well plates (Greiner Bio-one, Australia) for 4 d to yield biofilm mass of mean absorbance (SE) at OD_600__nm_ of 0.86 (0.05). The bacteriophage EFA1 significantly lowered the mean biofilm mass at all treatments from 2 to 24 h (*p* < 0.001) (Figure 5C). The mean (SE) biomass following treatment with EFA1 bacteriophage ranged from 0.47 (0.02) for a 2 h treatment to 0.6 (0.07) for a 24 h treatment. While there was a significant reduction in biofilm following treatments at all time points, exposure to EFA1 for 2 h resulted in the greatest decrease in biofilm mass, and the biofilm mass was shown to increase with the length of EFA1 treatment (*p* < 0.001). Paired *T*-tests revealed statistically increased mean (SE) absorbance of biofilm mass between EFA1 treatments for 2 h [0.47 (0.02)] and 6 h [0.52 (0.02)], 4 h [0.48 (0.02)] and 8 h [0.58 (0.02)]; 6 h [0.52 (0.02)] and 24 h [0.6 (0.07)], 8 h [(0.58 (0.02)], and 24 h [0.6 (0.07)]. In order to assess whether the bacteria was developing resistance to EFA1 with the longer treatments, a 5 days biofilm was exposed to EFA1 for (a) 24 h (b) 2 h, and (c) 24 h + 2 h (where after 24 h, media was replaced with fresh media and EFA1, then left for a further 2 h). In these experiments, the biofilm reduction with the 24 h + 2 h treatment was similar to that for 2 h, suggesting that *E. faecalis* had not developed resistance to EFA1 (Figure 5D).

### EFA1 Effect on the Proliferation and ROS Production of HCT116 Colon Cancer Cell Line Co-cultured With *E. faecalis*

#### HCT116 Colon Cancer Cell Proliferation in *E. faecalis* Co-culture

**FIGURE 6:**
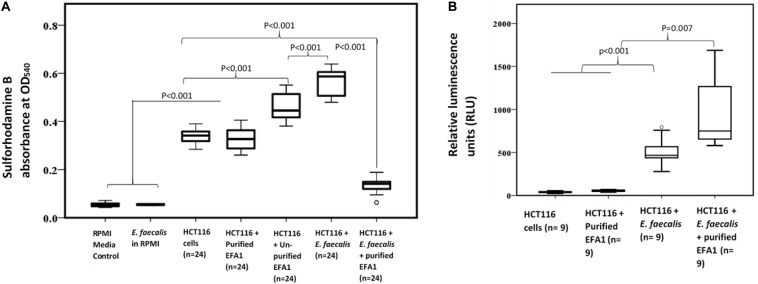
Bacteriophage EFA modulates the effects of *E. faecalis* on HCT116 cancer cell line by increasing ROS production and attenuating cancer cell proliferation. **(A)** Cancer cell proliferation was significantly increased in *E. faecalis* co-cultured HCT116 cells compared to all other cultures. The RPMI growth media and *E. faecalis* growing in RPMI showed minimal SRB absorbance. HCT116 cells grown with purified bacteriophage EFA1, and the HCT116 cells grown on their own, had similar levels of proliferation. Treatment of the HCT116 cells/*E. faecalis* co-culture with purified bacteriophage EFA1 resulted in significant reduction of cancer cell growth, to levels below those of the control HCT116 culture. **(B)** ROS production was significantly increased in the HCT116/*E. faecalis* co-culture with purified EFA1, and to a lesser extent in the HCT116/*E. faecalis* co-culture, compared to HCT116 cells on their own. There was no statistical difference in ROS production between untreated HCT116 and those grown with purified bacteriophage EFA1.

As SRB stoichiometrically binds to protein under mild acidic conditions, the absorbance of bound SRB is proportional to cell mass and hence used as proxy for cell proliferation (Orellana and Kasinski, 2016). The greatest proliferation of HCT116 colon cancer cells was seen when co-cultured with *E. faecalis* (Figure 6A). The crudely prepared bacteriophage EFA1 caused an increased proliferation of HCT116 cells with mean (SE) SRB absorbance of 0.46 (0.02), *p* < 0.001 (Figure 6A), while the mean (SE) SRB absorbance of purified EFA1 treated HCT116 colon cancer cells of 0.31 (0.02) was comparable to that of untreated cells: 0.34 (0.01) (*p* = 0.709). Both the untreated HCT116 cells and those treated with purified EFA1 had significantly less proliferation than HCT116 treatment with unpurified EFA1 and the *E. faecalis*/HCT116 colon cancer cell co-culture [0.57 (0.02), *p* < 0.001] (Figure 6A). The least cell proliferation was seen in HCT116 cells that were co-cultured with *E. faecalis* and purified bacteriophage EFA1 with mean (SE) SRB absorbance of 0.13 (0.02) equivalent to a 77.2% reduction in cell proliferation (*p* < 0.001) (Figure 6A). The RPMI growth media and *E. faecalis* growing in RPMI showed minimal SRB absorbance.

#### ROS Production in the *E. faecalis*/HCT116 Colon Cancer Cell Co-culture

To investigate the ROS production in co-culture, RLU as proxy for ROS production were compared between HCT116 colon cancer cells alone and those in co-culture with *E. faecalis* (Figure 6B). HCT116 colon cancer cells alone had similar RLU to HCT116 colon cancer cells with added purified bacteriophage EFA1 (*P* = 0.200). The median (Q1–Q3) RLU for HCT116 cells and the HCT116 with EFA1 were 45.2 (31.2–106.1) and 59.5 (45.5–65.7), respectively. The HCT116 colon cancer cells that were co-cultured with *E. faecalis* had higher RLU than both non-treated HCT116 cancer cells and those with purified EFA1 (*P* < 0.001) (Figure 6B). The median (Q1–Q3) RLU for HCT116 co-cultured with *E. faecalis* was 467.0 (365.7–662.3). The RLU of HCT116 colon cancer cells co-cultured with *E. faecalis* and purified bacteriophage EFA1 was significantly increased over that of the HCT116 colon cancer cell pure culture, HCT116 colon cancer cells with EFA1 (*P* < 0.001), and HCT116 colon cancer cells co-cultured with *E. faecalis* (*p* = 0.007) (Figure 6B). The mean (Q1-Q3) RLU of the HCT116 colon cancer cells co-cultured with *E. faecalis* and bacteriophage EFA1 was 748.8 (643.8–1475.1).

## Discussion

In this study we describe the isolation and characterization of the lytic bacteriophage EFA1 which targets *E. faecalis*. We also describe its modulation of the growth stimulating effects of *E. faecalis* in a colon cancer cell co-culture. There have not been any other studies investigating the actions of *Enterococcus* bacteriophages in colon cancer cells and *E. faecalis* bacterial co-cultures. EFA1 belongs to the family *Siphoviridae*. The bacteriophage host range did not extend to the other bacterial species tested. As such, EFA1 has the potential to minimize impact on other microbes if used in a diverse microbial environment. In terms of its replication kinetics, the bacteriophage had a latency of 20 min, and a burst size of 120 PFU/bacterial cell. This compares to a latency of 30 min and burst size of 116 for the *E. faecium* bacteriophage IME-Efm1 (Wang et al., 2014), which is the closest related bacteriophage whose replication kinetics are known. To date there are at least 80 *Enterococcus* bacteriophage genomic sequences deposited in NCBI GenBank (Accessed 31 July 2020). The diversity among these viruses is very broad. EFA1 is most closely related to the *E. faecalis* bacteriophage Santor1. These two are part of a monophyletic group of 11 bacteriophages which target *E. faecalis* or *E. faecium*, and which also form a larger clade with bacteriophages infecting *Lactobacillus* and *Streptococcus* hosts, but which cluster more distantly from other bacteriophages which target *E. faecalis* hosts. The genomic arrangement and predicted putative genes found in the bacteriophage EFA1 genome infers a lytic bacteriophage lifestyle and therefore supports its potential suitability to be used in therapy. This was further supported by the lack of toxins or factors that may enhance bacterial virulence such as antibiotic resistance (Mathew, 2016). Some bacteriophages have been shown to carry antibiotic resistant genes that may be transferred between biomes (Muniesa et al., 2013). While genomic analysis of EFA1 did not show any putative genes coding for such bacterial virulence enhancing proteins, ORF33 in EFA1 coded for a putative beta-lactamase protein. However, amino-acid alignment with the bacterial beta-lactamase sequence showed very little identity between the two proteins. Further, screening with the antibiotic resistance CARD database did not show any positive hits for putative antibiotic resistance genes (Jia et al., 2017) in the EFA1 genome. The InterProScan (Mitchell et al., 2019) and Prosite (Sigrist et al., 2013) did not reveal any active sites or beta-lactamase domains required for catalysis (Moali et al., 2003) in the EFA1 genome either. While the ORF 33 genetic element is currently apparently non-functional as a beta-lactamase, it may have allowed the transfer of *E. faecalis* antibiotic resistance by bacteriophages in the past. ORF33 is located among genes coding for putative DNA manipulation, and it is possible its function is in this area, or otherwise unknown, rather than that of a bacterial beta-lactamase. Most of the *Enterococcus* bacteriophages that form a clade with EFA1 also contain a genetic element with some similarity to ORF 33, and so this may represent an evolutionary link between closely related viruses.

Functionally, bacteriophage EFA1 was capable of disrupting *E. faecalis* biofilms within 2 h of treatment. This was evidenced using confocal microscope imaging that revealed sparse cells on bacteriophage treated biofilm compared to untreated biofilm. Quantification of the biofilm mass supported these findings. With increased exposure to the bacteriophage, the biofilm mass began to steadily increase. However, it is likely that this consistent increase was not due to bacterial resistance to EFA1, as adding fresh media and EFA1 after 24 h resulted in biofilm reduction similar to that seen after exposure for 2 h. In this connection, studies with the *E. coli* bacteriophage T4 in *E. coli* mono-biofilms revealed increased bacterial growth after prolonged treatment from 4 to 6 h (Corbin et al., 2001). This phenomenon was not seen in planktonic cultures but considered to be unique to biofilms and suggested to be important in maintenance of the bacteria and bacteriophage ecosystem (Sutherland et al., 2004). These findings raise important issues with respect to potential applications of bacteriophages such as EFA1 in therapy. For instance, they highlight the need for further experiments to more precisely define these host-parasite interactions and kinetics, so that timing of bacteriophage treatment regimens results in optimal biofilm degradation.

Since *E. faecalis* has been shown to be closely linked to CRC, we investigated the effect of this bacteria on the proliferation of HCT116 colon cancer cells. Similar to the effects of oncobacteria such as *F. nucleatum* (Rubinstein et al., 2013, 2019), *E. faecalis* increased the proliferation of HCT116 colon cancer cells. We found that when we exposed HCT116 cells to unpurified EFA1 (which did not have treatment to rid remnant lipoteichoic acid and/or other cell debris), there was a mitogenic effect and stimulation of cancer cell growth. This effect was lost when EFA1 was purified to remove bacterial cell debris. When treated with purified bacteriophage EFA1, *E. faecalis* induced HCT116 colon cancer cell proliferation was significantly attenuated to levels below that of untreated HCT116 colon cancer cells. To investigate this further we assayed for ROS production. In HCT116 cells co-cultured with *E. faecalis* there was a significant increase, while exposure of HCT116 to purified EFA1 alone resulted in no change, in ROS levels. Our findings also showed significantly increased ROS production, to levels higher than in the HCT116/*E. faecalis* co-culture, when purified bacteriophage EFA1 was added to the HCT116/*E. faecalis* co-culture. We could not find any reports investigating the effects of bacteriophage and associated bacteria on ROS production in cancer cells or mammalian cells other than in leukocytes (Przerwa et al., 2006; Miedzybrodzki et al., 2008; Miernikiewicz et al., 2013). In these studies, there was a decrease in ROS when bacteriophage T4 treated granulocytes were co-cultured with *E. coli*, in contrast with our findings. Therefore, it may be that this difference in ROS induction is dependent on cell type.

The role of ROS in cancer (Liou and Storz, 2010) and in cancer therapy (Trachootham et al., 2009) has been reviewed. It is known that cells express antioxidants that neutralize ROS while maintaining sufficient levels needed for cellular signaling (Gonzalez et al., 2002). In some tumorigenic events, increased intracellular ROS promotes tumor progression (Jackson, 1994). However, high and excessive ROS levels are induced by chemotherapy agents leading to cell cycle arrest and cell death (Yang H. et al., 2018). Although the exact mechanism and effect of ROS production in our system is unknown, and requires further investigation, the excessive ROS production could contribute to the significantly lower cancer cell proliferation seen when the HCT116/*E. faecalis* co-culture was treated with EFA1.

## Conclusion

The bacteriophage EFA1 isolated from wastewater was fully characterized and its whole genome sequenced. EFA1 was capable of disrupting *E. faecalis* mono-biofilms. Further, while *E. faecalis* enhanced the proliferation of HCT116 colon cancer cells, in the presence of EFA1 the proliferation was significantly inhibited with an associated increase in ROS production. EFA1 alone did not significantly alter the proliferation or ROS production of HCT116 cells. Such findings may lead to further testing of bacteriophages such as EFA1 in the control of oncobacteria induced cancer cell growth.

## Data Availability Statement

The authors declare that all relevant data supporting the findings of the study are available in this article and the Supplementary Figure file, or from the corresponding author upon reasonable request. The complete genome sequence of bacteriophage EFA1 has been submitted to NCBI GenBank under accession number MT857001.

## Author Contributions

JT and MK: conceptualization, data analysis, and writing—original draft. MK and TM-A: mammalian cell and bacterial co-culture. MK and SB: genomic. MK and HK: electron microscope imaging and biofilm. JT, HC, and SP: supervision. All authors contributed to writing, reviewing, and editing of the manuscript.

## Conflict of Interest

The authors declare that the research was conducted in the absence of any commercial or financial relationships that could be construed as a potential conflict of interest.

## Footnotes

1 http://www.geneious.com/
