# Supplementary material for: Lytic Bacteriophage EFA1 Modulates HCT116 Colon Cancer Cell Growth and Upregulates ROS Production in an Enterococcus faecalis Co-culture System
Source: Front Microbiol. 2021 Mar 31;12:650849. doi: 10.3389/fmicb.2021.650849 (PMC8044584; doi:10.3389/fmicb.2021.650849)
Supplement: Supplementary file 1 [file Data_Sheet_1.PDF]

Supplementary Material

Table S1: Bacteriophage genome annotation and predicated Open reading Frames

ORF	Coordinates	Size (aa)	Significant match	% Identity	Query cover (%)	E0 value
orf1	97..231	44	hypothetical protein [Enterococcus phage vB_EfaS_Ef5.4]	81%	97%	9.00E-17
orf2	197..343	48	hypothetical protein Max_01 [Enterococcus phage vB_EfaS_Max]	83.78%	77%	2.00E-12
orf3	270..422	50	hypothetical protein [Enterococcus phage IME_EF3]	98%	100%	8.00E-28
orf4	437..910	157	terminase small subunit [Enterococcus phage EFAP-1]	100.00%	100%	5.00E-112
orf5	1140..1256	38	hypothetical protein [Enterococcus phage vB_EfaS_Ef5.4]	92.11%	100%	2.00E-15
orf6	1446..3170	575	terminase large subunit [Enterococcus phage IME-EF4]	100.00%	99%	0.00E+00
orf7	3201..3404	67	hypothetical protein phiSHEF4_59 [Enterococcus phage phiSHEF4]	98.51%	100%	5.00E-37
orf8	3409..4560	383	portal protein [Enterococcus phage phiSHEF5]	97.91%	100%	0.00E+00
orf9	4523..5110	195	prohead protease [Enterococcus phage EFRM31]	97.95%	100%	1.00E-135
orf10	5174..6430	418	capsid family protein [Enterococcus phage phiSHEF4]	97.12%	99%	0.00E+00
orf11	6555..6758	67	hypothetical protein [Enterococcus phage vB_EfaS_Ef5.4]	95.45%	98%	8.00E-37
orf12	6794..7096	101	head-tail joining protein [Enterococcus phage EFRM31]	100%	99%	2.00E-66
orf13	7068..7403	111	head-tail adaptor protein [Enterococcus phage EFRM31]	99.10%	100%	3.00E-75
orf14	7400..7807	135	head-tail joining protein [Enterococcus phage EFRM31]	99.26%	100%	2.00E-95
orf15	7804..8169	121	head-tail joining protein [Enterococcus phage EFRM31]	99.17%	100%	1.00E-84
orf16	8248..8808	186	major tail protein [Enterococcus phage phiSHEF4]	100%	100%	1.00E-129
orf17	8971..9297	108	putative tail tape measure chaperone protein [Enterococcus phage EfaCPT1]	99.03%	95%	3.00E-67
orf18	9554..13924	1456	tail length tape-measure protein [Enterococcus phage SANTOR1]	96.77%	100%	0.00E+00
orf19	14006..16081	691	tail protein [Enterococcus phage phiSHEF4]	97.83%	100%	0.00E+00
orf20	16126..18354	742	tail assembly protein [Enterococcus phage SANTOR1]	97.84%	100%	0.00E+00
orf21	18535..18780	82	hypothetical protein [Enterococcus phage IME_EF3]	100%	98%	9.00E-50
orf22	19027..20013	328	autolysin [Enterococcus phage phiSHEF2]	97.87%	100%	0.00E+00
orf23	complement (20094..20321)	76	glutaredoxin [Enterococcus phage vB_EfaS_Max]	92.00%	98.00%	2.00E-45
orf24	complement (20322..20918)	198	DNA modification [Enterococcus phage vB_EfaS_Ef5.2]	99.49%	100%	3.00E-143
orf25	complement (20981..23272)	763	putative DNA polymerase [Enterococcus phage EfaCPT1]	94.63%	100%	0.00E+00
orf26	complement (23307..23537)	77	hypothetical protein [Enterococcus phage vB_EfaS_Ef5.4]	100.00%	96.00%	1.00E-45
orf27	complement (23602..24309)	236	hypothetical protein [Enterococcus phage vB_EfaS_Ef5.1]	98.72%	99.00%	8.00E-168
orf28	complement (24387..24635)	83	hypothetical protein SANTOR1_0125 [Enterococcus phage SANTOR1]	100.00%	98%	3.00E-52
orf29	complement (24636..24932)	99	hypothetical protein phiSHEF4_19 [Enterococcus phage phiSHEF4]	96.94%	98.00%	7.00E-64
orf30	complement (24933..25751)	273	hypothetical protein [Enterococcus phage vB_EfaS_Ef5.3]	98.90%	99.00%	0.00E+00
orf31	complement (25741..25977)	79	hypothetical protein vBEfaSAL2_33 [Enterococcus phage vB_EfaS_AL2]	93.65%	79.00%	9.00E-35
orf32	complement (25913..26077)	55	hypothetical protein [Enterococcus phage IME_EF3]	85.19%	98.00%	8.00E-25
orf33	complement (26050..26865)	272	beta-lactamase superfamily domain protein [Enterococcus phage phiSHEF4]	96.64%	98.00%	0.00E+00
orf34	complement (26819..27382)	188	NUMOD4 motif protein [Enterococcus phage phiSHEF4]	98.84%	91.00%	4.00E-121
orf35	complement (27349..27828)	160	putative NHN homing endonuclease-like protein [Enterococcus phage phiNASRA1]	97.48%	99.00%	1.00E-111
orf36	complement (27825..28061)	79	hypothetical protein SANTOR1_0150 [Enterococcus phage SANTOR1]	98.53%	86.00%	5.00E-41
orf37	complement (28033..28632)	200	hypothetical protein [Enterococcus phage PMBT2]	95.98%	99.00%	2.00E-139
orf38	complement (28794..29006)	71	hypothetical protein [Enterococcus phage Nonaheksakonda]	94.29%	98.00%	3.00E-43
orf39	complement (29003..29740)	246	putative prim-pol domain protein [Enterococcus phage EfaCPT1]	99.18%	99.00%	5.00E-180
orf40	complement (29752..29940)	63	hypothetical protein IME-EF4_26 [Enterococcus phage IME-EF4]	98.39%	98.00%	7.00E-36
orf41	complement (29999..30175)	59	hypothetical protein phiSHEF4_30 [Enterococcus phage phiSHEF4]	96.55%	98.00%	1.00E-30

orf42	complement (30172..31482)	437	putative helicase [Enterococcus phage EfaCPT1]	92.31%	98%	0.00E+00
orf43	complement (31460..32017)	186	HNH endonuclease family protein [Enterococcus phage EFRM31]	64.12%	91.00%	1.00E-68
orf44	complement (31950..32321)	124	hypothetical protein SANTOR1_0190 [Enterococcus phage SANTOR1]	88.89%	94.00%	4.00E-61
orf45	complement (32293..32526)	78	hypothetical protein [Enterococcus phage vB_EfaS_Ef5.2]	100.00%	98.00%	2.00E-46
orf46	complement (32529..32747)	73	hypothetical protein [Enterococcus phage LY0322]	98.55%	94.00%	5.00E-41
orf47	complement (32740..32901)	54	hypothetical protein IME-EF4_21 [Enterococcus phage IME-EF4]	100.00%	98.00%	8.00E-27
orf48	complement (32903..33175)	91	hypothetical protein [Enterococcus phage vB_EfaS_IME196]	97.78%	98.00%	1.00E-53
orf49	complement (33224..33667)	148	hypothetical protein EfaCPT1_gp44 [Enterococcus phage EfaCPT1]	95.21%	98.00%	1.00E-99
orf50	complement (33741..33929)	63	hypothetical protein [Enterococcus phage vB_EfaS_IME196]	87.10%	98.00%	1.00E-29
orf51	complement (33967..34317)	117	DUF1140 protein [Enterococcus phage EfaCPT1]	76.92%	88.00%	1.00E-49
orf52	complement (34392..34580)	63	hypothetical protein [Enterococcus phage vB_EfaS_Ef5.1]	100.00%	98.00%	1.00E-34
orf53	complement (34670..36250)	527	DNA primase/helicase [Enterococcus phage PMBT2]	98.86%	99%	0.00E+00
orf54	complement (36346..36543)	66	putative membrane protein [Enterococcus phage vB_EfaS_LM99]	100.00%	98.00%	3.00E-34
orf55	complement (36540..36779)	80	hypothetical protein [Enterococcus phage vB_EfaS_Ef5.2]	97.47%	98.00%	2.00E-48
orf56	complement (36776..36991)	72	hypothetical protein phiSHEF2_52 [Enterococcus phage phiSHEF2]	91.55%	98.00%	1.00E-39
orf57	complement (37003..37206)	68	hypothetical protein [Enterococcus phage phiNASRA1]	98.51%	98.00%	9.00E-42
orf58	complement (37203..37415)	71	transcriptional regulator [Enterococcus phage phiNASRA1]	100.00%	98.00%	8.00E-43
orf59	complement (37415..37666)	84	hypothetical protein ZZ2_047 [Enterococcus phage Ec-ZZ2]	87.95%	98.00%	7.00E-44
orf60	complement (37639..37779)	47	hypothetical protein [Enterococcus phage vB_EfaS_IME196]	91.30%	97.00%	8.00E-24
orf61	complement (37773..37937)	55	hypothetical protein ZZ2_046 [Enterococcus phage Ec-ZZ2]	96.30%	98.00%	8.00E-31
orf62	complement (37949..38056)	36	hypothetical protein [Enterococcus phage vB_EfaS_IME196]	89.66%	80%	4.00E-09
orf63	complement (38093..38353)	87	hypothetical protein LM99_0057 [Enterococcus phage vB_EfaS_LM99]	100.00%	98.00%	8.00E-57
orf64	complement (38392..38757)	122	hypothetical protein [Enterococcus phage vB_EfaS_Ef5.3]	90.91%	99.00%	8.00E-39
orf65	complement (38762..38980)	73	hypothetical protein [Enterococcus phage vB_EfaS_Ef5.3]	79.17%	98.00%	2.00E-32
orf66	39491..39694	67	putative membrane protein [Enterococcus phage vB_EfaS_LM99]	86.57%	100%	2.00E-33
orf67	39777..39983	69	hypothetical protein SANTOR1_0295 [Enterococcus phage SANTOR1]	100.00%	98.00%	1.00E-41
orf68	39971..40354	128	hypothetical protein SANTOR1_0300 [Enterococcus phage SANTOR1]	100.00%	96.00%	8.00E-88
